# Spatiotemporal Immunomodulation Using Biomimetic Scaffold Promotes Endochondral Ossification‐Mediated Bone Healing

**DOI:** 10.1002/advs.202100143

**Published:** 2021-03-16

**Authors:** Yutong Liu, Zhaogang Yang, Lixuan Wang, Lili Sun, Betty Y. S. Kim, Wen Jiang, Yuan Yuan, Changsheng Liu

**Affiliations:** ^1^ Key Laboratory for Ultrafine Materials of Ministry of Education School of Materials Science and Engineering, and Engineering Research Center for Biomedical Materials of Ministry of Education East China University of Science and Technology Shanghai 200237 P. R. China; ^2^ Department of Radiation Oncology University of Texas Southwestern Medical Center Dallas TX 75390 USA; ^3^ Department of Neurosurgery The University of Texas MD Anderson Cancer Center Houston TX 77030 USA

**Keywords:** dexamethasone, endochondral ossification, hypoxia signaling pathway, immunomodulation, sequential release

## Abstract

Biomaterials play an important role in treating bone defects by promoting direct osteogenic healing through intramembranous ossification (IO). However, majority of the body's bones form via cartilaginous intermediates by endochondral ossification (EO), a process that has not been well mimicked by engineered scaffolds, thus limiting their clinical utility in treating large segmental bone defects. Here, by entrapping corticosteroid dexamethasone within biomimetic recombinant human bone morphogenetic protein (rhBMP)‐loaded porous mesoporous bioglass scaffolds and regulating their release kinetics, significant degree of ectopic bone formation through endochondral ossification is achieved. By regulating the recruitment and polarization of immune suppressive macrophage phenotypes, the scaffold promotes rapid chondrogenesis by activating Hif‐3*α* signaling pathway in mesenchymal stem cells, which upregulates the expression of downstream chondrogenic genes. Inhibition of Hif‐3*α* signaling reverses the endochondral ossification phenotype. Together, these results reveal a strategy to facilitate developmental bone growth process using immune modulating biomimetic scaffolds, thus providing new opportunities for developing biomaterials capable of inducing natural tissue regeneration.

## Graphical abstract

A spatiotemporal dexamethasone‐entrapped, rhBMP‐2‐loaded porous mesoporous bioglass scaffold is fabricated to precisely regulate developmental endochondral ossification. The initial release of dexamethasone can effectively mediate the inflammatory response involving M2/M1 ≈ 1 before endochondral stage, and further promotes later rhBMP‐2‐induced endochondral ossification via rapid chondrogenesis by activating Hif‐3*α* signaling pathway and ensuing ossification by hypertrophy of chondrocytes, angiogenesis, and osteogenesis.

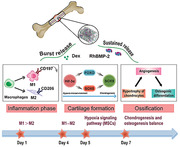

## 1 Introduction

Bone fractures cause disability worldwide, leaving many patients in pain and discomfort. Although therapeutic treatments have improved in recent decades, delayed healing and nonunion commonly occur in the clinic, sparking the interest of researchers to find better treatments. Knowing the complex and sequential steps of bone fracture healing is a necessary prerequisite to advance clinical treatment.^[^ ^1^ ^]^ Generally, bone formation often occurs in two distinct steps: intramembranous ossification (IO) and endochondral ossification (EO).^[^ ^2^, ^3^ ^]^ By analyzing animal and clinical data, the predominant importance of the EO‐based bone fracture healing process has become increasingly evident from a clinical standpoint, especially for healing large segmental bone defects.^[^ ^4^, ^5^, ^6^ ^]^ EO is characterized by differentiating condensed mesenchymal cells into chondrocytes, proliferation, and hypertrophy of chondrocytes, and further vascularization and mineralized bone formation. The whole process is mainly regulated by Ihh/PTHrp signaling, fibroblast growth factor (FGF) signaling, and bone morphogenetic protein (BMP) signaling.^[^ ^3^, ^7^, ^8^ ^]^ From a biomimetic and clinical perspective, constructing advanced bone substitutes inspired by the EO mechanism is a potent strategy for promoting large bone fracture healing. However, so far, previously published biomaterials for bone regeneration have mainly focused on the IO process; relatively less literature to date has reported EO‐based bone regeneration process. Hence, from developmental viewpoint, in‐depth investigations of bone substitutes to induce the EO process are greatly in need.^[^ ^9^ ^]^

Immune responses knowingly accompany the implantation of bone biomaterials both in IO and EO. And, EO‐based bone healing occurred mainly with three sequential stages, involving inflammatory stage (from day 1 to day 5), endochondral stage (from day 5 to day 21) and remodeling stage (from day 21 to day 35).^[^ ^10^ ^]^ Many previous reports have proven that disadvantageous inflammation responses such as systemic activation of polymorphonuclear neutrophils (PMNs) and acute inflammation with LPS treatment can result in impaired fracture healing.^[^ ^11^ ^]^ Therefore, regulating immune responses by controlling immune cell behavior at initial stages is currently considered a promising strategy to develop advanced bone biomaterials. Specifically, macrophages are known among various kinds of immune cells for their quick recruitment and long‐lived residue at regenerative sites.^[^ ^1^ ^]^ Besides their phagocytic function, macrophages are known for high flexibility and ability to polarize to M1 (pro‐inflammation) and M2 (anti‐inflammation) phenotypes to the surrounding microenvironment.^[^ ^12^, ^13^ ^]^ In a typical in vivo scenario, after biomaterial implantation, M1 macrophages are rapidly initiated and predominated,^[^ ^12^ ^]^ and subsequent transitioning from M1 to M2 phenotype is associated with a series of signal transductions and is widely acknowledged as being essential for tissue regeneration.^[^ ^14^ ^]^ Therefore, to achieve a desirable microenvironment, M1 or M2 phenotypes have been regulated from various viewpoints, including surface topology and chemical compositions of biomaterials, cytokines, and drugs.^[^ ^15^, ^16^ ^]^ But for the EO process, only one study reported that M2 macrophages facilitated fracture healing through it.^[^ ^16^ ^]^ Furthermore, how to precisely and timely immunomodulate biomaterials for efficient EO‐based bone regeneration is still unclear.

**Figure 1:**
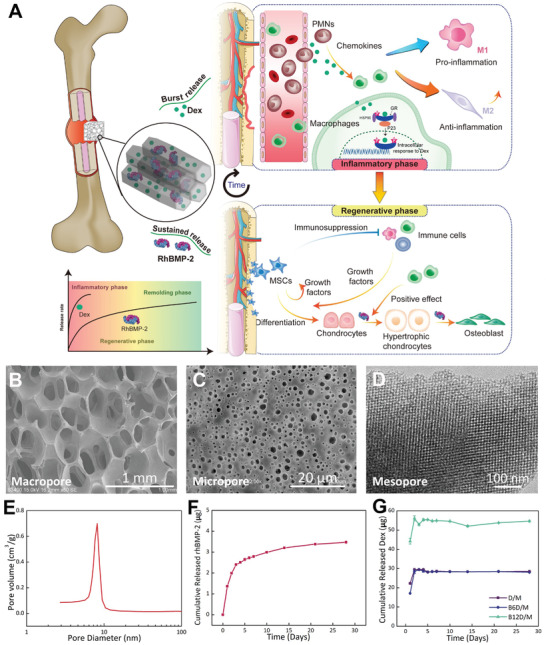
Schematic diagram of the experimental design and characterizations of sequential release system. A) Scheme of designed multiporous tunnels for sequential release of Dex and rhBMP‐2. A burst release of Dex aims to precisely modulate an appropriate inflammatory response in the early stage, and thus promotes the sustained‐release rhBMP‐2‐induced EO bone regeneration. SEM images of macroporous structure B) and microporous structure C). D) TEM image of mesoporous structure. E) Mesopore size distribution. F) Release curve of rhBMP‐2. G) Release curves of Dex.

As one of important signaling pathways for EO, BMP signaling is well‐accepted to mainly facilitate mesenchymal condensation and to increase proliferation of chondrocytes in the later stage.^[^ ^8^ ^]^ Therefore, we decided to explore the feasibility of using the anti‐inflammatory drug dexamethasone (Dex) to regulate early immune responses and thus boost an EO process in this study. To achieve this, a rhBMP‐2‐loaded hierarchical macro/micro/nanoporous mesoporous bioglass (MBG) scaffold, a typical biomimetic bone substitute associated with highly osteogeneration capacity via EO process, was chosen as the model matrix.^[^ ^4^ ^]^ Dex was spatiotemporally incorporated to accurately modulate the inflammatory microenvironment through glucocorticoid receptor (GR).^[^ ^17^, ^18^ ^]^ We hypothesized that by controlling the loading amount and rapid release of Dex, this system could effectively modulate the early‐stage (in 5 days postimplantation) inflammatory responses, and subsequently accelerated the rhBMP‐2‐induced endochondral bone formation mainly involving MSCs recruitment and differentiation in the later stage (**Figure** **1**A). Considering the similarity between ectopic and orthotopic bone formation in the initial stage of endochondral ossification, as well as experimental operability, an ectopic model was adopted to study the EO process.^[^ ^19^ ^]^ We examined macrophages recruitment and polarization at different time points. We also investigated ectopic bone formation and endochondral ossification through histological analysis and symbolic characteristics. To explore the underlying mechanisms, we measured MSCs recruitment, differentiation, and RNA‐Seq.

## 2 Results

### 2.1 Fabrication of BD/M Scaffolds and Sequential Release of Dex and rhBMP‐2

Inspired by the sequential steps of bone fracture healing, we selected hierarchical MBG scaffolds to construct a sequential delivery system of Dex and rhBMP‐2 (recombinant human bone morphogenetic protein‐2) to match the requirement of various stages (Figure 1). RhBMP‐2 was entrapped into the mesopores and Dex was physically adsorbed into the MBG scaffolds. As expected, the MBG scaffolds prepared here exhibited an interconnected macroporous structure with a pore size of 200–500 µm (Figure 1B). Micropores (1–4 µm), which is defined as pores with micrometer size (<10 µm), were uniformly distributed across the surface of the macropore walls with higher magnification (Figure 1C).^[^ ^4^ ^]^ Transmission electron microscopy (TEM) images presented a nanometric pore canal within the MBG frame wall (Figure 1D). The average pore diameter of the mesopores measured by Barrete–Joynere–Halenda (BJH) analyses was 8.2 nm, a comparable size with the rhBMP‐2 molecule (7 × 3.5 × 3 nm^3^) (Figure 1E).

Next, we immobilized rhBMP‐2 using a “saturated volume adsorption” method followed by the adsorption of Dex. Intensity peaks of B/M (rhBMP‐2/MBG) were lower in SAXRD than D/M (Dex/MBG) and M (MBG) (Figure S1, the Supporting Information), indicating the entry of rhBMP‐2 into the mesopores of MBG scaffolds. A burst release of 27.32% rhBMP‐2 occurred after 24 h followed by a sustained release through which rhBMP‐2 (69.49%) was released at 4 weeks (Figure 1F). According to our previous studies, released rhBMP‐2 maintained its native structure and osteogenic efficacy.^[^ ^4^, ^5^ ^]^ The release curves of Dex showed an initial burst release of immobilized Dex of about 90% over 48 h both in B6D/M (rhBMP‐2 6Dex/MBG, rhBMP‐2: Dex at mass ratio of 1:6) and B12D/M (rhBMP‐2 12Dex/MBG, rhBMP‐2: Dex at mass ratio of 1:12), followed by adsorption and desorption dynamic equilibrium (Figure 1G). The dosage of the incorporated Dex was taken carefully into consideration by the immunomodulatory efficacy exhibited with higher Dex loadings (30 and 60 µg) (Figure S2, the Supporting Information) together with our previous clinic results that the rhBMP‐2: Dex at mass ratio of 1:6 achieved the best bone regeneration efficacy. As mentioned earlier, the sequential release of rhBMP‐2 and Dex was applied utilizing hierarchical MBG scaffolds.

### 2.2 Histology, Immunohistochemistry, and Immunofluorescence

**Figure 2:**
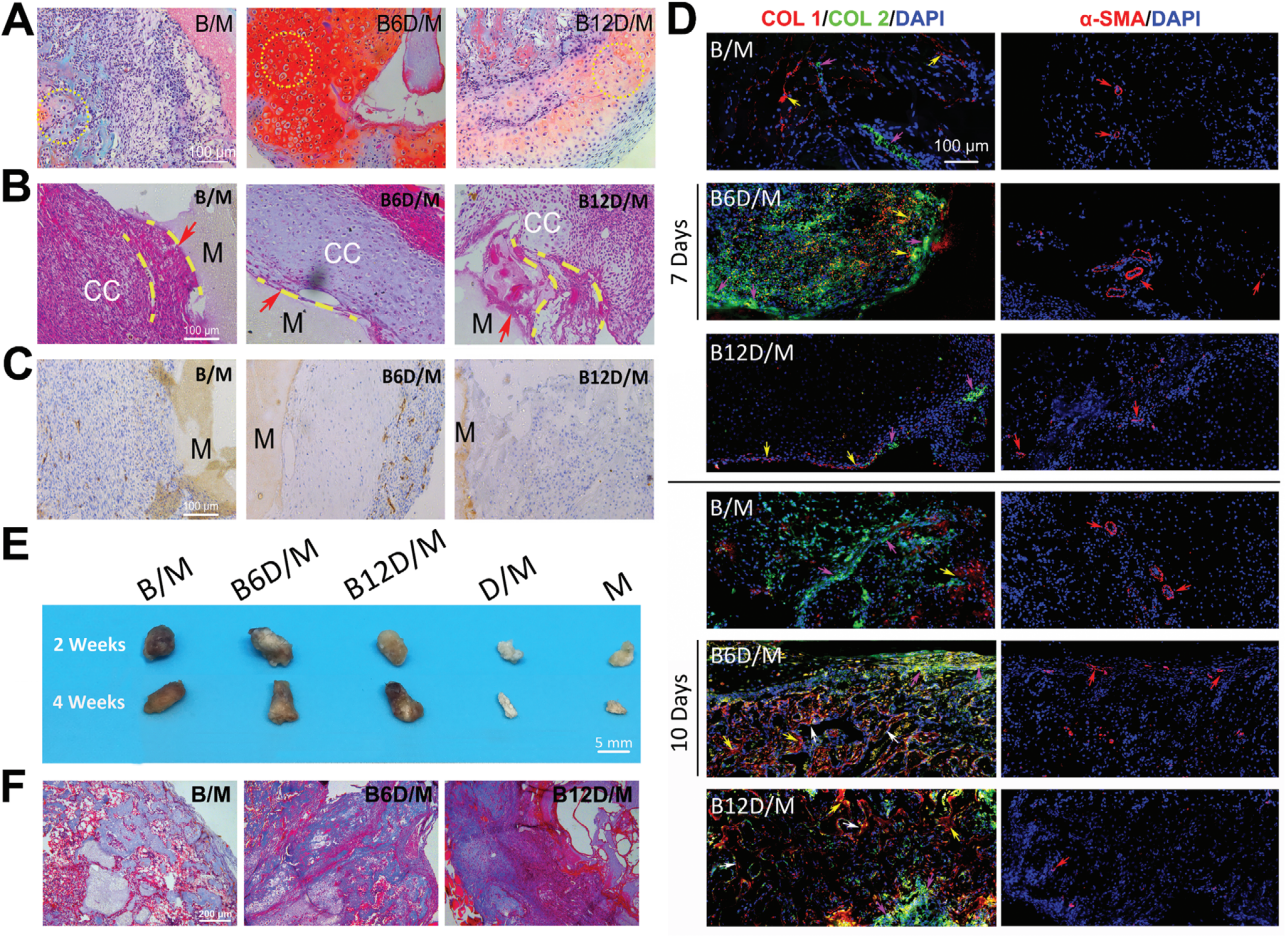
In vivo chondrogenesis, vascularization, and ossification. A) Safranin‐O‐fast green staining at 7 days. The expression of aggrecan was visualized in red (yellow circle). B) HE staining at 7 days. (Interface between implanted material and tissue: red arrow; surrounding fibrous: yellow dash lines; cartilage: CC; woven bone: WB; implanted material: M.) C) Immunohistochemistry analyses of CD31 expression at 7 days. D) Immuno‐fluorescence analysis of COL 1 (red), COL 2 (green), and *α*‐SMA (red) at 7 and 10 days; and nucleus stained with DAPI (blue). (COL 1: yellow arrow; COL 2: pink arrow; vascular: red arrow; mature bone: white arrow.)) E) Digital images of ectopic bones at 2 and 4 weeks. F) Masson's trichrome staining of B/M, B6D/M and B12D/M at 2 weeks.

We carefully examined the EO process and osteogenic capacity of BD/M scaffolds. First, BD/M samples were retrieved after 7 days of implantation and stained with Safranin‐O‐fast green to visualize cartilage formation (**Figure** **2**A). B6D/M showed the maximum and most extensive cartilage area of all groups. To further identify endochondral ossification, samples were observed after HE staining (Figure 2B; Figure S3A, the Supporting Information). At 7 days, conspicuous fibers were observed between the implanted materials and newly formed cartilages in B/M and B12D/M (shown as yellow dashed lines), indicating an undesired inflammatory response mainly due to improper macrophages behaviors and inadequate integration between materials and tissues. In contrast, a few fibers were shown in B6D/M and thick newly formed cartilages were adjacent to the B6D/M scaffold. At day 10, proximal cartilages and distalis woven bones were seen in B6D/M and B12D/M. Nevertheless, a mass of fibers still remained in B/M. Cartilages and woven bones were evident in all groups at this time. Besides, B6D/M exhibited the most extensive CD31 expression during the period of endochondral ossification (Figure 2C; Figure S3B, the Supporting Information). Interestingly, the infused vessels visualized by CD31 in B6D/M were adjacent to newly formed cartilages. Histological analysis showed a coherent distribution of material, cartilages, and capillary vessels in B6D/M at 7 days.

Furthermore, immunofluorescence analysis was conducted to identify the expression of osteogenic (COL 1 (red)), chondrogenic (COL 2 (green)), and vascular (*α*‐SMA (red)) marker, with the nucleus stained with DAPI (blue) (Figure 2D; Figure S3C, the Supporting Information). At 7 days, the most abundant and adjoining distribution of COL 2 and dotted COL 1 protein were observed in B6D/M. At day 10, shown as the morphology exhibited with DAPI‐labeled nucleus (blue) and intensive expression of COL 1 (red), widespread mature woven bone was observed in B6D/M and B12D/M (white arrow), while the expression of COL 1 and COL 2 was relatively higher in B/M than at day 7. At each time point, the expression of *α*‐SMA was shown in all groups, indicating successful vascular invasion, an essential step for ossification.

Osteogenic efficacy of BD/M scaffolds was determined following a typical EO process as verified with the above results. The digital pictures and histograms intuitively presented the facades and sizes of ectopic bone formation at 2 and 4 weeks (Figure 2E; Figure S4B, the Supporting Information).^[^ ^4^, ^5^ ^]^ Apparently, no ectopic bone formation was induced by D/M and M scaffolds. The ectopic bone formation amounts induced by B6D/M scaffolds were higher than those induced by with B/M and B12D/M scaffolds at 2 weeks; they were lower but without significant difference in all groups at 4 weeks because of bone resorption.

HE and Masson's trichrome staining confirmed no bone formation induced by D/M and M scaffolds; the pictures showed many fibers and none woven bones (Figure 2F; Figure S4C,D, the Supporting Information). At 2 weeks, woven bone tissue fulfilled the implanted sites of B/M, B6D/M, and B12D/M. B6D/M groups showed the most extensive and thickest woven bones at 2 weeks, as revealed by both HE and Masson's trichrome staining. The amplificatory Masson's trichrome staining pictures evidenced a typical EO process in B6D/M and B12D/M groups, as hypertrophic chondrocytes were found to adjoin newly formed woven bones. At 4 weeks, fat vacuoles partially replaced woven bones in all groups in virtue of bone resorption. Specifically, less fat vacuoles were observed in B6D/M groups, which indicated enhanced and prolonged efficacy of rhBMP‐2. Based on the above, B6D/M facilitated and induced higher amount of ectopic bone formation through the EO process.

### 2.3 In Vivo Recruitment of F4/80+ Monocytes/Macrophages

**Figure 3:**
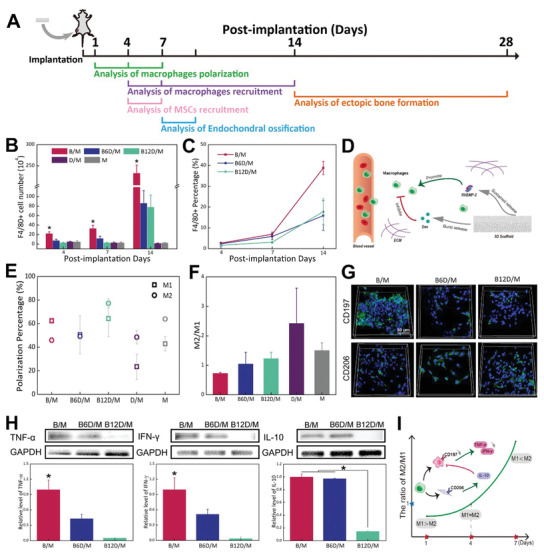
Immunomodulation effect of Dex on F4/80+ monocytes and macrophages recruitment and macrophages phenotypes. A) Experimental design time flowchart. B) Quantification of F4/80+ cell number. C) Percentage of F4/80+ cells. D) Scheme of recruited macrophages to implantation. Dex inhibited macrophages recruitment from surrounding tissues and blood vessels while rhBMP‐2 promoted them. Recruited macrophages could be customized with the proper ratio of Dex and rhBMP‐2. E) Percentages of M1 phenotype and M2 phenotype of five groups at 4 days. F) Ratio of M2/M1 of B/M, B6D/M, B12D/M, D/M and M at 4 days. G) Immunofluorescence images of CD197 and CD206 expression (green), and nucleus stained with DAPI (blue). H) Protein expression of TNF‐*α*, IFN‐*γ*, and IL‐10. I) Ratio of M2/M1 in B6D/M at different time points. M1 macrophages predominated at day 1 and M2 macrophages predominated at day 7. Intriguingly, the ratio of M2/M1 approached 1 at day 4 in B6D/M, with significantly lower M1 macrophages and pro‐inflammatory cytokines than B/M. (*p < 0.05, *N* = 3.)

Obvious weakened fibrosis was shown in B6D/M throughout the EO process, which was greatly influenced by infiltration and polarization of macrophages.^[^ ^20^ ^]^ Moreover, macrophages that are recruited to regenerative sites exert vital effect on the whole EO process.^[^ ^1^ ^]^ Thus, we investigated Dex's effect on macrophages penetration after scaffold implantation. At 4, 7, and 14 days, we observed through flow cytometry that F4/80+ monocytes/macrophages were recruited to implanted sites (**Figure** **3**B,C; Figure S5A, the Supporting Information). The minimum amounts of F4/80+ monocytes/macrophages were shown in D/M and M with no significant differences in numbers.

Next, B/M and BD/M scaffolds were analyzed carefully considering the bone formation process. Herein, the number of F4/80+ monocytes/macrophages in each group increased as bone formation evolved and the incorporation of Dex greatly decreased recruited F4/80+ monocytes/macrophages cell numbers as expected. At day 4, the number of recruited F4/80+ monocytes/macrophages were 22.18 ± 3.78 × 10^4^ by B/M, 7.33 ± 2.92 × 10^4^ by B6D/M and 3.01 ± 0.72 × 10^4^ by B12D/M. At day 7 and 14, the number of recruited F4/80+ monocytes/macrophages into B6D/M scaffolds was approximately one‐third of those observed in the B/M scaffolds, and recruited cells were further lowered by B12D/M (Figure 3B). According to the results, rhBMP‐2 promoted sustained macrophage recruitment while burst‐released Dex inhibited excessive macrophage recruitment in the early stages, highlighting the importance of the rhBMP‐2 and Dex ratio (Figure 3D).

### 2.4 Effect of Dex on Macrophages Phenotypes In Vivo

M1 and M2 macrophages play different roles in bone formation. To explore the Dex‐mediated early inflammatory response, different macrophage phenotypes (CD197 for M1 and CD206 for M2) were determined at days 1, 4, 7 (Figure S5C, the Supporting Information). At each time point, the M2/M1 ratio was increased by incorporating Dex, revealing the function of downregulated inflammation by Dex (Figure S5D, the Supporting Information). Meanwhile, the M2/M1 ratio increased over time in all groups, indicating the transformation from pro‐inflammation to proregeneration. The highest polarization ratio of macrophages was specifically found at day 4 (Figure S5C, the Supporting Information). Therefore, we studied the macrophage phenotypes of five groups at day 4 postimplantation (Figure 3E,F). D/M represented the highest M2/M1 ratio, but the lowest macrophage polarization. B6D/M and B12D/M improved the M2/M1 ratio in contrast to B/M (0.74 ± 0.02 M2/M1 ratio). Conspicuously, Dex incorporated with a 6 ratio of rhBMP‐2 reduced the M1 phenotype from 62.4% to 50.53% and mildly increased the M2 phenotype from 45.9% to 49.28%, in comparison with B/M. At 4 days, M1 and M2 phenotypes of B6D/M were present in a similar percentage (50.53% ± 16.16% M1 phenotype and 49.28% ± 2.47% M2 phenotype). The B12D/M groups further facilitated the transition of macrophages toward the M2 phenotype (77.2% M2 phenotype). Additionally, cell surface markers (shown in green) were determined by immunofluorescent staining (Figure 3G). Images showed that CD197 expression was lower with B6D/M than with B/M. TNF‐*α*, IFN‐*γ*, and IL‐10 expression also indicated that B6D/M downregulated the pro‐inflammatory response in early stages because TNF‐*α* and IFN‐*γ* levels were significantly lower than B/M, and IL‐10 protein expression of both groups was similar (Figure 3H). Protein expressions of TNF‐*α*, IFN‐*γ*, and IL‐10 were almost suppressed by B12D/M, highlighting an oversuppressive function by adding extra Dex. Overall, appropriate Dex lowered M1 macrophage numbers but had a minimal effect on M2 macrophages, as shown by the significant downregulation of pro‐inflammatory cytokines (Figure 3I), and a close percentage of M1 and M2 macrophages were realized in B6D/M after 4 days.

### 2.5 Recruitment and Differentiation of MSCs onto BD/M Scaffolds

**Figure 4:**
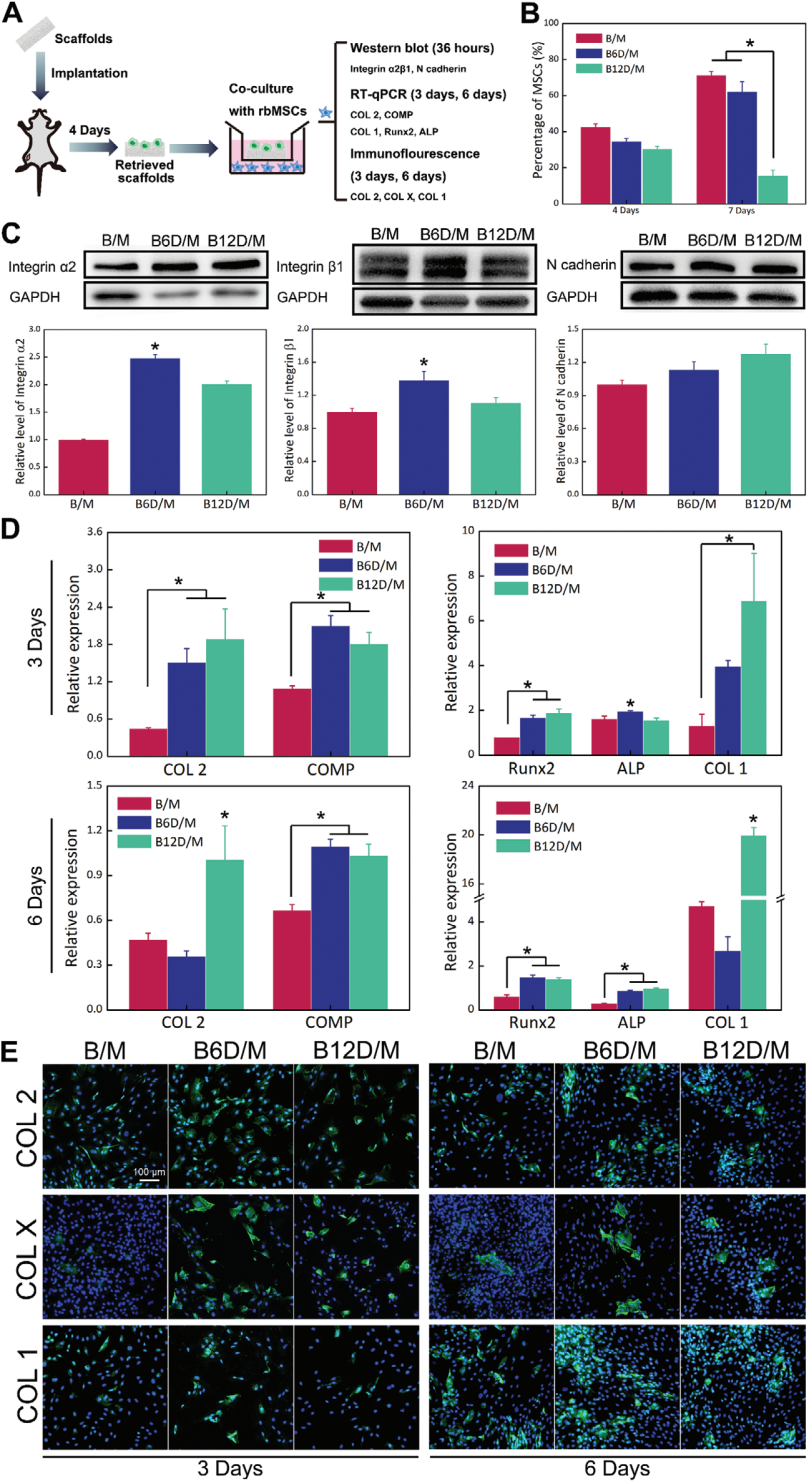
Recruitment and differentiation of MSCs in BD/M scaffold‐mediated immune microenvironment. A) A scheme for co‐culture experiments. B) Percentage of recruited MSCs by flow cytometry. C) Protein expression of Integrin *α*2*β*1 and N cadherin. D) Gene expression of COL 2, COMP (chondrogenetic marker), and Runx2, ALP, COL 1 (osteogenetic marker). E) Immunofluorescence images of COL 1, COL 2, and COL *X* expression (green); and cellular nuclei stained with DAPI (blue). (*p < 0.05, *N* = 3.)

As mentioned earlier, early inflammation response was shown to directly exert effect on ensuing MSCs response, including MSCs recruitment and condensation.^[^ ^21^ ^]^ Therefore, we first pinpointed the changing of MSCs recruitment by flow cytometry at 4 and 7 days after implantation (**Figure** **4**B; Figure S6, the Supporting Information). After 4 days, the percentages of recruited MSCs in B6D/M (35.04% ± 1.20%) and B12D/M (30.88% ± 1.06%) were similar to those for B/M (42.98% ± 1.38%). Notably, the recruited MSCs were much less in B12D/M (15.99% ± 2.76%) than in other groups after 7 days, while the recruited MSCs were higher in B/M (71.56% ± 1.80%) and B6D/M (62.41% ± 5.41%).

To reveal the correlation between the immune microenvironment and differentiation of MSCs, ex vivo co‐culture experiments were performed utilizing a trans‐well system. After transplanting 4 days, a well‐chosen time point with different ratios of M2/M1 in experimental groups and with little dosage of Dex, the retrieved samples were placed in the upper inserts and cultured with rbMSCs. After incubating for 36 h, the intramembrane protein expression was examined by Western blot analysis (Figure 4C). Integrin *α*2*β*1 and N cadherin, which are essential for cartilage formation, were significantly enhanced in B6D/M but not in B/M. Chondrogenic and osteogenic differentiation of rbMSCs were determined by RT‐qPCR and immunofluorescence staining. After co‐culturing for 3 days, gene expression of COL 2, COMP (chondrogenic marker), Runx2, and COL 1 (osteogenesis marker) were significantly enhanced in B6D/M and B12D/M. After co‐culturing for 6 days, the relative gene expression of COL 2 and COMP was less than or equal to GAPDH (house‐keeping gene), while the relative levels of Runx2 and ALP were heightened in B6D/M and B12D/M (Figure 4D). These results indicate the attenuation of chondrogenesis over time.

Furthermore, protein expression of COL 2, COL *X*, and COL 1 was visualized by immunofluorescence analysis (Figure 4E; Figure S7A, the Supporting Information). After co‐culturing for 3 days, COL 2 and COL *X* were intuitively enhanced in rbMSCs by B6D/M and B12D/M, whereas the expression of COL 1 was inconspicuous in all groups. Intriguingly, an obvious breakdown was shown in COL *X* expression and a prominent increase was shown in COL 1 expression at 6 days. Based on the results mentioned above, the EO process was perfectly replicated with the trans‐well system ex vivo and promoted by B6D/M.

### 2.6 Mechanism of B6D/M‐Induced Initial Chondrogenesis

**Figure 5:**
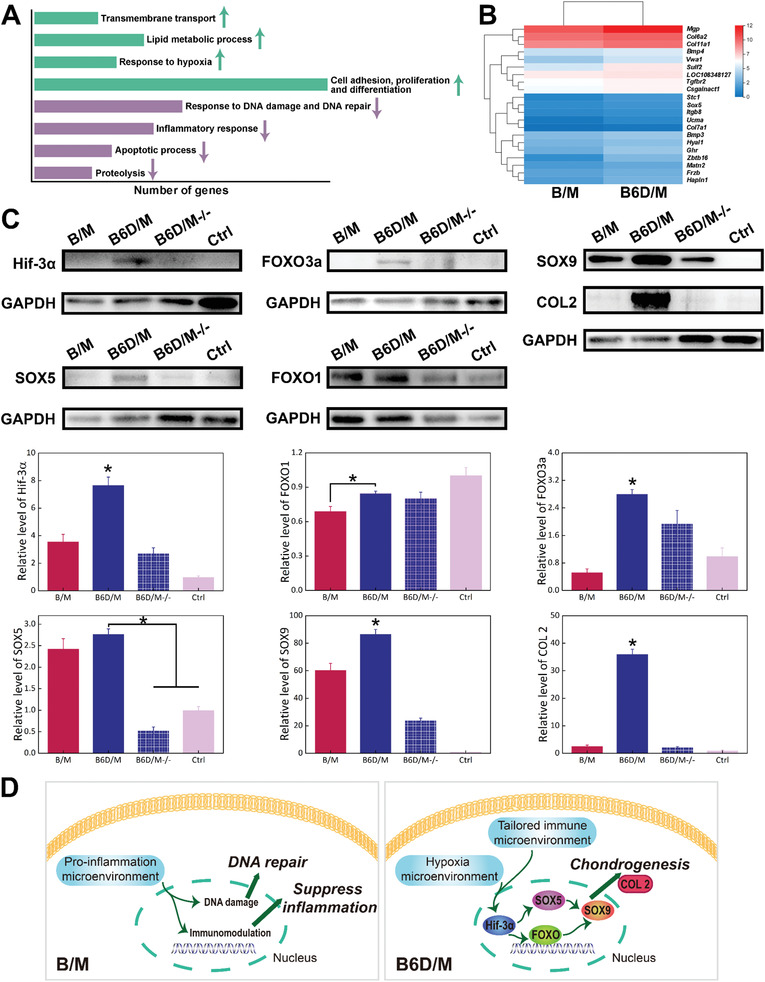
Underlying mechanism of B6D/M‐facilitated chondrogenesis by RNA‐Seq. A) The number of significantly regulated genes (≥2‐fold difference: upregulated (green arrow) and downregulated (purple arrow)) for bone formation and immune biological process. B) A heatmap of significantly differentially expressed mRNA levels related to chondrogenesis. C) Protein expression of Hif‐3*α*, SOX5, FOXO1, FOXO3a, SOX9, COL 2 analyzed by Western blotting. D) Different cell behaviors on B/M and B6D/M. Pro‐inflammation microenvironment in B/M‐initiated DNA damage and immunomodulation capability. Tailored immune microenvironment by B6D/M activated Hif‐3*α*, thereafter stimulated FOXO signaling pathway and SOX5, then upregulated SOX9, and ultimately initiated the development of COL 2 indicating rapid chondrogenic differentiation of MSCs. (*p < 0.05, *N* = 3.)

Considering the unique comparable M2/M1ratio at 4 days and ensuing facilitated EO of B6D/M, the intracellular response of rbMSCs stimulated with different immune microenvironments in B/M and B6D/M after day 4 postimplantation was further studied. RbMSCs were incubated with the retrieved B/M and B6D/M scaffolds from the 4‐day implantation and then analyzed by RNA‐Seq. The heatmap revealed 882 downregulated genes, 1092 upregulated genes, and differentially expressed mRNA level by B6D/M in comparison with B/M (Figure S8A, the Supporting Information). Upregulated and downregulated KEGG pathway is illustrated in **Figure** **5**A. The results revealed that the B6D/M group upregulated transmembrane transport, lipid metabolic process, response to hypoxia and cell adhesion, and cell proliferation and differentiation. Meanwhile, DNA damage and DNA repair, inflammatory response, apoptotic process, and proteolysis were obviously more weakened in B6D/M than in B/M. The significantly upregulated chondrogenic genes are shown in Figure 5B. Specially, Hif‐3*α* was significantly upregulated in B6D/M among genes responding to hypoxia. Moreover, FOXO signaling pathway, SOX5 and SOX9 were upregulated in B6D/M in comparison with B/M (Figure S8C, the Supporting Information). As cartilage development occurred in hypoxia microenvironment, we hypothesized that Hif‐3*α* should play a vital role in early‐stage chondrogenic differentiation of MSCs. Figure 5C confirmed that B6D/M indeed enhanced the expressions of Hif‐3*α*, SOX5, FOXO1, FOXO3a, SOX9, COL 2. After Hif‐3*α* was blocked with Hif‐3a siRNA, the protein expression of SOX5, FOXO3a, SOX9, and COL 2 in B6D/M−/− were significantly downregulated compared to B6D/M (Figure 5C).

Based on the above results, the untailored pro‐inflammation microenvironment in B/M initiated a cellular response to DNA damage and triggered the immunomodulatory capability of MSCs, leading to DNA repair and inflammation suppression. In contrast, B6D/M induced the proper immune microenvironment and stimulated hypoxia signaling pathway. More precisely, the activation of Hif‐3*α* facilitated FOXO signaling pathway and SOX5, ensuing upregulated SOX9, and ultimately promoted the development of COL 2, leading to rapid chondrogenic differentiation of MSCs (Figure 5D).

## 3 Discussion

In most clinical cases, bone fracture healing occurs through EO with sequential steps including chondrogenic differentiation of MSCs, proliferation and hypertrophy of chondrocytes, and coupled invasion of osteoblasts and vessels. The inflammatory stage, the first stage of bone fracture healing, is widely recognized as being critical for initiating EO that involves immune cells and related cellular factors. Macrophages not are only essential contributors throughout the inflammatory stage,^[^ ^12^, ^22^ ^]^ but also exhibit extraordinary plasticity toward M1 and M2 phenotypes. Although many studies have shown the positive effect of M2 macrophages on IO and EO, ambiguity of the timing and accurate regulation of the inflammatory stage and its effect on EO still creates a bottleneck for experimental studies and clinical treatment. Inspired by these previous studies, we proposed to apply a common clinical anti‐inflammatory drug, Dex, to accurately immunomodulate the EO process.

In order to precisely regulate the inflammatory stage, which mainly take place in the initial 5 days as reported,^[^ ^10^ ^]^ the dosage and release rate of Dex were carefully tailored. With rhBMP‐2/MBG (B/M) as a matrix, various doses of Dex were incorporated according to the efficient performance of modulating macrophage infiltration and phenotypes (Figure S2, the Supporting Information), and Dex was initially and quickly released in 2 days. We thought that the BD/M scaffolds with various Dex amounts mainly regulated inflammatory microenvironment at different levels in the early stage, and thus exerted an effect on B/M‐induced bone regeneration. Meanwhile, given the complexity of the orthotopic bone formation in vivo, and higher cost and larger defect area of the surgery, we selected an ectopic model to study the effects of the early‐stage inflammatory response on late‐stage endochondral ossification. Ectopic models are known to exhibit similar endochondral ossification in the early stages with orthotopic bone formation. As anticipated, ectopic bone formation visualized with digital images and histology was significantly improved by B6D/M scaffolds at 2 weeks (Figure 2E,F; Figure S4, the Supporting Information). Because an obvious difference was shown in groups with and without Dex, and 90% Dex was released in 2 days (Figure 1H), we hypothesized an important function of early inflammation responses occurring in 7 days in EO.

EO‐based fracture healing is initiated by the early inflammatory response (Figure 1A). Inadequate and prolonged inflammation responses often result in impaired and delayed bone regeneration,^[^ ^1^, ^23^ ^]^ and inflammation is complex and involves a cascade of immune cells at the regenerative site. PMNs recruit to the fracture site and attract macrophages by secreting several chemokines.^[^ ^24^ ^]^ After macrophages have completed their function, lymphocytes recruit and initiate adaptive inflammation.^[^ ^25^ ^]^ Compared to D/M and M, the increased number of F4/80+ monocytes/macrophages in the BD/M scaffolds confirmed macrophages’ indispensable role in ectopic bone formation, as previously reported.^[^ ^16^, ^26^, ^27^ ^]^ Furthermore, our results indicated that the Dex‐loaded groups could effectively suppress excessive macrophage infiltration (Figure 3B,C; Figure S5A, the Supporting Information) and decreased the M1 (pro‐inflammation) population in the first stage. At first sight, M1 macrophages dominated initially and M2 macrophages increased in all groups (Figure S5C,D, the Supporting Information). This transition from the M1 to M2 population corresponded to the inflammation phase transitioning to the regeneration phase, in accordance with previous studies.^[^ ^14^, ^16^ ^]^ Elaborate insight into our results showed that the percentages of the M1 and M2 population were highest at day 4 (Figure 3E,F; Figure S5B, the Supporting Information), which revealed an active behavior of the macrophages at this time. Furthermore, almost percentages of the M1 and M2 subtypes were evidently represented in the B6D/M group at day 4, which represented a balanced immune microenvironment. Also, B6D/M definitely downregulated the presence of M1 macrophages and barely affected M2 macrophages when compared to B/M, which was confirmed by the protein expression levels of TNF‐*α*, IFN‐*γ*, and IL‐10, and also immunofluorescence images of the retrieved samples (Figure 3G,H). Considering the function of M2 macrophage in osteogenesis,^[^ ^16^, ^28^ ^]^ our results highlight that a balanced M1/M2 population and a corresponding timely and appropriate immune response was more favorable for excellent bone fracture healing in vivo. These outcomes emphasized a critical and balanced role of both M1 and M2 populations excited by B6D/M at day 4, a critical time point before cartilage formation.

The EO process ideally requires optimal cartilage formation and successful transition from cartilage to bone, thus a chondrogenic/osteogenic balance hypothesis was previously proposed by our group.^[^ ^5^ ^]^ Our results showed no significant difference in the recruited MSCs in all groups at day 4 and recruited MSCs were greatly increased in B/M and B6D/M at day 7, but MSCs recruitment was lower in B12D/M at day 7 (Figure 4B; Figure S6, the Supporting Information). As MSCs recruitment to implanted site was driven by chemokines secreted by immune cells, our results indicated that the inflammation responses stimulated by rapid‐released Dex would exert effect on the ensuing MSCs recruitment. Also the over anti‐inflammatory microenvironment by B12D/M hampered infiltration of MSCs to the implanted site due to inadequate pro‐inflammatory factors for MSCs recruitment.^[^ ^27^ ^]^ Also, B6D/M was more favorable for the upregulation of chondrogenic‐ and osteogenic‐related genes and proteins (Figure 4D,E; Figure S7A, the Supporting Information). Particularly, an increase of Runx2, an important osteogenic transcription factor for chondrocyte hypertrophy,^[^ ^29^ ^]^ and COL *X* protein expression, were observed in B6D/M. Moreover, the relatively attenuated chondrogenic related genes, together with the downregulation of COL *X* and upregulation of COL 1, from day 3 to day 6, indicated a chondrogenesis/osteogenesis balance. These results were confirmed by abundant vessels invaded from the peripheral area to the thick layer of cartilage in B6D/M at day 7 (Figure 2C,D). Also, the initiation of ossification with the coupled invaded osteoblasts, as well as oxygen and nutrition transported by vessels is in a similar manner to that of orthotopic bone regeneration.^[^ ^27^, ^30^ ^]^ More specifically, a coherent distribution of material, cartilages, and capillary vessels was shown in histology analysis, indicated perfect biointegration under adequate immune microenvironment by B6D/M. Based on these results, we conclude that a typical EO was recapitulated by B6D/M for harmonious bone regeneration. Moreover, because EO is a requirement for hematopoietic stem cell (HSC) niche formation, a perfect remolding phase could be predicted.^[^ ^31^ ^]^ As for the B/M scaffold, belated transition from pro‐inflammatory macrophages to proregenerative macrophages resulted in relatively downregulated chondrogenic and osteogenic differentiation of MSCs. Moreover, a mass of fibrous tissue separated from the newly formed cartilage and B/M scaffold (Figure 2B), indicating that excessive macrophages infiltration and insufficient M2 macrophages in the early stage inhibited the integration between tissues and materials. In contrast, overdepression of MSCs recruitment by B12D/M after day 4 led to insufficient motivation of ensuring the EO process (Figure 2 and 4B).

**Scheme 1:**
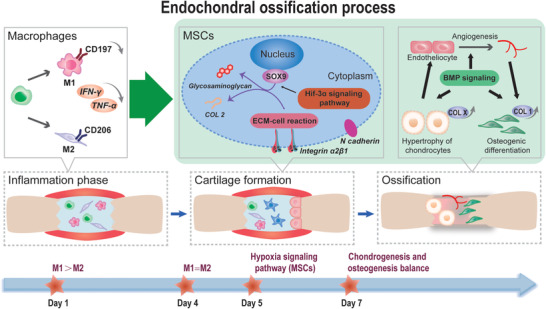
B6D/M‐recapitulated endochondral ossification process. Dex promoted EO mainly including three well‐acknowledged phases: inflammation, cartilage formation, and ossification. Specifically, 1) the initial inflammation phase. With a burst release of adequate dosage of Dex, a transition of the ratio of M2/M1 macrophages from <1 to 1 occurred on B6D/M at 4 days, right before the initiation of cartilage formation. 2) Cartilage formation (4–7 days). B6D/M facilitated ECM‐cell reaction, and activated the Hif‐3*α* signaling pathway and downstream SOX9 in MSCs, leading to rapid cartilage formation. 3) In the late stage, owing to great implant integration as a result of early‐stage proper immune microenvironment, B6D/M boosted BMP‐based endochondral ossification phase, including hypertrophy of chondrocytes, angiogenesis, and osteogenesis.

This noticeable Dex‐mediated appropriate immune environment and ensuing facilitated EO process drew our attention. According to our results and to those of previous reports, three explanations are possible (**Scheme** **1**). The first factor taken into account is the function of transmembrane proteins, which is responsible for the interaction between cells and extracellular matrix. N cadherin is essential for initiating MSC condensation and negatively affects the Wnt/*β* catenin signaling pathway,^[^ ^32^, ^33^ ^]^ while Integrin *α*2*β*1 favors cartilage matrix condensation.^[^ ^34^ ^]^ Therefore, the upregulation of both Integrin *α*2*β*1 and N cadherin should contribute to early‐stage MSCs and cartilage matrix condensation under the unique immune microenvironment of B6D/M (Figure 4C). The second factor is that the B6D/M‐induced immune microenvironment stimulated the hypoxia signaling pathway, which is crucial for chondrogenesis differentiation, chondrocytes proliferation, and, therefore cartilage enlargement. Specifically, B6D/M activated Hif‐3*α* signaling pathway and its downstream FOXO signaling pathway and SOX5 (Figure 5C; Figure S8C, the Supporting Information), which greatly upregulated SOX9, a key chondrogenic transcription factor, and consequently promoted the chondrogenesis of MSCs. Hif‐3*α* was previously reported to contribute to stable chondrocytes phenotypes.^[^ ^18^ ^]^ The FOXO signaling pathway was recently reported to facilitate the expression of SOX 9,^[^ ^35^ ^]^ a key chondrogenic transcription factor and SOX5 was acknowledged as an upstream transcription factor of SOX9. But, disappointingly, the pro‐inflammation microenvironment in B/M probably resulted in DNA damage in MSCs, activated DNA repair related genes and led to incapable chondrogenic initiation (Figure 5A,D). The third factor is related to the B6D/M‐induced immune microenvironment facilitated vascularization, and boosted the sustained‐release rhBMP‐2‐mediated EO process (Figure 2D). BMP signaling is well‐accepted to achieve EO by mesenchymal condensation and chondrocytes proliferation of in the later stage.^[^ ^8^ ^]^ What's more, our results indicated that B6D/M did favor the vascularization (Figure 2C,D), which is considered to play a critical role in endochondral ossification and bone remodeling during late EO process.

As we considered implant integration, MSCs recruitment, and chondrogenic/osteogenic balance based on transmembrane proteins, hypoxia signaling pathway, and BMP signaling, the results here indicated that balanced M1 and M2 macrophage phenotypes by B6D/M at day 4 in the ectopic site recapitulated a perfect EO process. Using an immunoregulatory strategy that exploits a cheap and common anti‐inflammation drug, our work highlights the clinical and commercial potential of B6D/M in EO‐based fracture healing.

## 4 Conclusion

We propose a precise Dex‐mediated immunomodulatory strategy that promotes the EO process for bone regeneration. Considering clinical application, rhBMP‐2‐loaded hierarchical porous MBG scaffolds were chosen to construct a biomaterial model. Dex was immobilized for quick initial release according to the sequential steps of bone formation. Specifically, balanced M1 and M2 macrophages excited by B6D/M at day 4 activated the Hif‐3*α* in MSCs, which stimulated FOXO signaling pathway and SOX5, facilitated expression of SOX9 and consequential COL 2, and hence rapidly induced cartilage formation. Overall, B6D/M favored implant integration, MSC recruitment and condensation, and chondrogenic/osteogenic balance. Prolonged inflammation in B/M led to massive fibrous tissue among implants and newly formed cartilage. Over suppression of inflammation responses caused adverse effects on MSCs recruitment and insufficient motivation to initiate EO. These results suggested that timely and appropriate modulation of early inflammatory stage was crucial to the EO process, which is applicable to bone fracture healing and other tissue regeneration.

## 5 Experimental Section

##### Preparation and Characterization of Hierarchical MBG Scaffolds

Hierarchical MBG scaffolds were fabricated by a modified multitemplate method, as previously reported.^[^ ^4^, ^36^ ^]^ Briefly, 4.0 g of P123, 6.70 g of tetraethyl orthosilicate (TEOS), 0.96 g of Ca(NO_3_)_2_·4H_2_O, 0.28 g of triethylphosphate (TEP), and 1.0 mL of HCl (0.5 m) were dissolved in 48 g ethanol and stirred at 30 °C for 24 h, followed by vacuum evaporation at 60 °C to obtain a viscous MBG sol. Polymer microspheres were mixed with the MBG sol at the mass ratio of 1:3. Trimodal MBG scaffolds (0.2 × 0.2 × 0.4 cm^3^) were fabricated with the polyurethane (PU) foam templating method. Thereby, a polyurethane sponge with a given shape was impregnated into a hybrid slurry until the sponge framework was completely coated with the hybrid slurry. After drying in the oven at 60 °C for 48 h, the samples were calcinated at 600 °C (heating rate at 1 °C·min^−1^) for 6 h to obtain the hierarchical MBG scaffold. The mesoporous, microporous, and macroporous structures of the MBG scaffold were characterized by high‐resolution transmission electron microscopy (HRTEM, JEM‐2010, JEOL, Japan) and field emission scanning electron microscope (FE‐SEM, Hitachi S‐4800, Japan). The pore diameter of the mesoporous strut was measured by BJH analysis (Tristar 3000, Micromeritics, USA).

##### Immobilization and Release of rhBMP‐2 and Dex on/from BMG Scaffolds

RhBMP‐2 (Shanghai Rebone Biomaterials Co., Ltd.) was immobilized in the mesopores of the MBG scaffold using the “saturated volume adsorption” method, as reported.^[^ ^4^ ^]^ Briefly, saturated volume of rhBMP‐2 solution was carefully dropped on the sterilized MBG scaffolds and vacuum‐freeze‐dried at −40 °C overnight. 5 µg of rhBMP‐2 were applied per scaffold. The rhBMP‐2/MBG (B/M) scaffolds were preserved at 4 °C for 1 h for full adsorption, followed by vacuum freeze drying at −60 °C overnight.

To prepare a composite rhBMP‐2/Dex/MBG (BD/M) scaffold, Dex (Chenxin pharmacy Co., Ltd.) was added to B/M scaffolds at different mass ratios with rhBMP‐2 at 1:6 (B6D/M) and 1:12 (B12D/M). Exactly, 30 and 60 µg of Dex were applied to each scaffold. Besides, 30 µg of Dex were added to the MBG scaffolds to prepare the Dex/MBG (D/M) composite scaffolds. Both D/M and MBG scaffold (M) were used as the control group. To facilitate the narrative, B/M, B6D/M, and B12D/M are collectively referred to as BD/M scaffolds.

In vitro Dex releasing profiles of three groups including B6D/M, B12D/M, and D/M were evaluated.^[^ ^37^ ^]^ Scaffolds were placed into tubes and immersed in 2 mL PBS at 37 °C under constant vibration at 30 rpm. At each time point, 1 mL of the solution was collected and added to 1 mL PBS. The amount of released Dex was quantitatively analyzed using ultraperformance liquid chromatography (UPLC, Agilent, Germany). In vitro rhBMP‐2 release was determined using a human BMP‐2 ELISA kit at each time point. The results were averaged with three tested specimens in each group.

##### Surgical Procedure and Sampling of Ectopic Bone Formation

Ectopic bone defect in thigh muscle is a typical model for standard EO‐based bone formation process.^[^ ^19^ ^]^ Thus, ectopic bone formation of BD/M, D/M, and M scaffolds was studied by implanting scaffolds into thigh muscle pouches of mice. Thirty male C57BL/6 mice were randomly divided into five groups and prepared for surgery. At 2 and 4 weeks, animals were euthanized and implants were harvested to weigh for wet bone. All procedures were carried out after being approved by the Institutional Animal Care and Use Committee of National Tissue Engineering Center (Shanghai, China).

##### Flow Cytometry

Flow cytometry was used to detect macrophages recruitment and phenotypes. Samples were harvested from C57BL/6 mice at 1, 4, 7, and 14 days after implantation. Cells from BD/M, D/M, and M were detached by composite enzyme (50% trypsin and 50% collagenase) at 37 °C for 5 min. After washing with PBS twice, cells were resuspended with cell staining buffer and blocked by rat anti‐mouse CD16/32 (Biolegende, USA) at 4 °C for 5 min. Then, cells were stained with rat anti‐mouse F4/80 PE antibody for determining F4/80+ monocytes/macrophages, rat anti‐mouse CD197 Alexa Fluor 647 antibody (BD, USA) for determining M1 phonotype, and rat anti‐mouse CD206 Alexa Fluor 647 antibody (BD, USA) for determining M2 phonotype. The samples were analyzed on an Accuri C6 flow cytometer (BD, USA) and data were analyzed using the FlowJo workstation (Tree Star, USA).

At 4 and 7 days after implantation, BD/M samples were harvested to analyze recruitment of MSCs to implants by flow cytometry. Briefly, cells were detached and stained with rat anti‐mouse CD44 antibody (Biolegende, USA) and Alexa Fluor 647‐labeled goat secondary antibody to rat (CST, USA), Armenian hamster anti‐mouse CD29 antibody (Biolegende, USA) and FITC‐labeled goat secondary antibody to hamster (Biolegende, USA), and rat anti‐mouse CD45 PE antibody (Biolegende, USA). All samples were analyzed by the CytoFLEX Platform (Beckman, USA).

##### Western Blot Analysis

To examine inflammatory factors, samples were collected after BD/M scaffolds were implanted into thigh muscle pouches of mice for 4 days. Proteins were extracted by RIPA lysis buffer, including phenylmethanesulfonyl (1 × 10^−3^ m). The extracted proteins were treated with 15% SDS‐PAGE and transferred onto polyvinylidene fluoride membranes. After being blocked with blocking buffer (Beyotime, China) at room temperature, the samples were incubated with TNF‐*α*, IFN‐*γ*, and IL‐10 (Biolegende, USA) primary antibodies at 4 °C overnight and then with goat polyclonal secondary antibody to rat IgG‐H&L (HRP) (Biolegende, USA) for 90 min.

To study the expression of rbMSCs transmembrane proteins, after 36 h incubation with retrieved BD/M scaffolds, rbMSCs (harvested from femur bone marrow in SD rats) of all groups were lysed and the extracted proteins were analyzed with SDS‐PAGE/immunoblotting. The samples were incubated with Integrin *α*2, *β*1, and N cadherin (Abcam, USA) primary antibodies overnight followed by goat secondary antibody to rabbit IgG‐H&L (HRP) (Abcam, USA) for Integrin *α*2, *β*1, and goat secondary antibody to mouse IgG‐H&L (HRP) (CST, USA) for N cadherin.

To verify cellular mechanism, Hif‐3*α* was blocked with Rat Hif‐3*α* siRNA (Dharmacon, USA). After 24 h incubation with retrieved BD/M scaffolds, rbMSCs of all groups were lysed and the extracted proteins were analyzed with SDS‐PAGE/immunoblotting. Specifically, B6D/M−/− referred to rbMSCs that was blocked with rat Hif‐3*α* siRNA and incubated with retrieved B6D/M scaffolds. The samples were incubated with Hif‐3*α* (Abcam, USA), FOXO1 (CST, USA), FOXO3a (CST, USA), SOX5 (ThermoFisher, USA), SOX9 (Abcam, USA), and COL 2 primary antibodies overnight followed by goat secondary antibody to rabbit IgG‐H&L (HRP) (CST, USA). All samples were normalized with the GAPDH protein. Protein expression was detected by chemiluminescence using High‐sig ECL Western Blotting Substrate (Tanon, China) by the Automatic chemiluminescence image analysis system (Tanon 5200, China).

##### Immunofluorescence Analysis

Immunofluorescence analysis was used to visualize the phenotype of the macrophages recruited to the implanted scaffolds. The retrieved BD/M scaffolds were fixed with 2.5% glutaraldehyde solution at 4 days after implantation. After blocking with a 5% bull serum albumin solution at 4 °C overnight, the samples were incubated with rabbit anti‐CD197 and rabbit anti‐CD206 antibodies (Abcam, USA) at 4 °C overnight and stained with Alexa Fluor 488‐labeled goat secondary antibody to rabbit (Absin, China) for 90 min. To examine protein expression of rbMSCs, after culturing with retrieved BD/M scaffolds for 3 and 6 days, rbMSCs were fixed and incubated with rabbit anti‐COL 1, anti‐COL 2, and anti‐COL *X* antibodies (Abcam, USA) overnight and stained with Alexa Fluor 488‐labeled goat secondary antibody to rabbit (Absin, China) for 90 min. The samples were observed with a confocal laser scanning microscope (CLSM, A1, Nikon, Japan).

##### Gene Expression by RT‐qPCR

To investigate MSC differentiation, chondrogenic and osteogenic related genes were examined by RT‐qPCR. After incubation for 3 and 6 days, gene expression of MSCs was examined by a real‐time quantitative reserve transcription‐polymerase chain reaction (RT‐qPCR) system (Bio‐Rad, USA). Markers for chondrogenesis, COL 2 and COMP, and for osteogenesis, Runx2, ALP, and COL 1, were determined and normalized with the GAPDH gene. The relative expression level (fold change) was calculated with the Livak method using 2^ΔΔ^Ct. All experiments were performed in triplicate. Primer sequences used here are listed in Table S1, the Supporting Information.

##### RNA Sequencing Analysis

The total RNA of rbMSCs was extracted using trizol after culturing with retrieved B/M and B6D/M scaffolds for 1 day. A Nano Drop and Agilent 2100 bionanalyzer (Thermo Fisher Scientific, MA, USA) was used to qualify and quantify total RNA. After rRNA was removed, cDNA was generated from the purified mRNA and amplified by PCR. The PCR products were heated, denatured, and circularized by splint oligo sequence. The single strand circle DNA was formatted as the final library and amplified with phi29 (Thermo Fisher Scientific, MA, USA) to make DNA nanoballs that were loaded into patterned nanoarrays. Single end 50‐base reads were generated on a BGISEQ200 platform (BGI, Shenzhen, China) and data were analyzed with Dr. Tom.

##### Histological, Immunohistochemistry, and Immunofluorescence Analyses of Endochondral Ossification

At each time point, samples were retrieved and fixed with 4% paraformaldehyde and decalcified in 12.5% EDTA for 2 weeks. Then, 4.5 µm thick sections were sliced. At day 7 and 10 after implantation, BD/M samples were stained with Safranin‐O‐fast green staining and hematoxylin/eosin (HE). At 2 weeks, samples of BD/M, D/M, and M were stained with Masson's trichrome. For immunohistochemical analysis, the sections were incubated with rabbit anti‐CD31 antibody (Abcam, USA) and with goat secondary antibody to rabbit IgG‐H&L (HRP) (Abcam, USA). The sections were stained with DAB substrate and treated with hematoxylin. The pictures were purchased from Leica Microsystems (Leica Microsystems Inc., Germany).

BD/M sections were incubated with COL 1 (Abcam, USA), COL 2 (Abcam, USA) and *α*‐SMA (Servicebio, China) primary antibodies and with Alexa Fluor 488‐labeled goat secondary antibody mouse (Servicebio, China) and Cy3‐labeled goat secondary antibody rabbit (Servicebio, China). The samples were observed with the Pannoramic MIDI scanning system (3D Histech, Hungary).

##### Statistical Analysis

All data were exhibited with mean standard deviation (SD). The significance was analyzed using one‐way ANOVA and Tukey's post hoc test; *p* value <0.05.

## Conflict of Interest

The authors declare no conflict of interest.

## Supporting information

Supporting Information

## Data Availability

Data available on request from the authors.
