# Supplementary material for: Spatiotemporal Immunomodulation Using Biomimetic Scaffold Promotes Endochondral Ossification‐Mediated Bone Healing
Source: Adv Sci (Weinh). 2021 Mar 16;8(11):2100143. doi: 10.1002/advs.202100143 (PMC8188258; doi:10.1002/advs.202100143)
Supplement: Supplementary file 1 — Supporting Information [file ADVS-8-2100143-s001.pdf]

© 2021 Wiley-VCH GmbH

ADVANCED SCIENCE
Open Access

Supporting Information

for *Adv. Sci.,* DOI: 10.1002/advs.202100143

Spatiotemporal immunomodulation using biomimetic scaffold promotes endochondral ossification-mediated bone healing

Yutong Liu, Zhaogang Yang, Lixuan Wang, Lili Sun, Betty Y. S. Kim, Wen. Jiang, Yuan Yuan*, and Changsheng Liu**

Supporting Information

Spatiotemporal immunomodulation using biomimetic scaffold promotes endochondral ossification-mediated bone healing

Yutong Liu, Zhaogang Yang, Lixuan Wang, Lili Sun, Betty Y. S. Kim, Wen. Jiang, Yuan Yuan*, and Changsheng Liu**

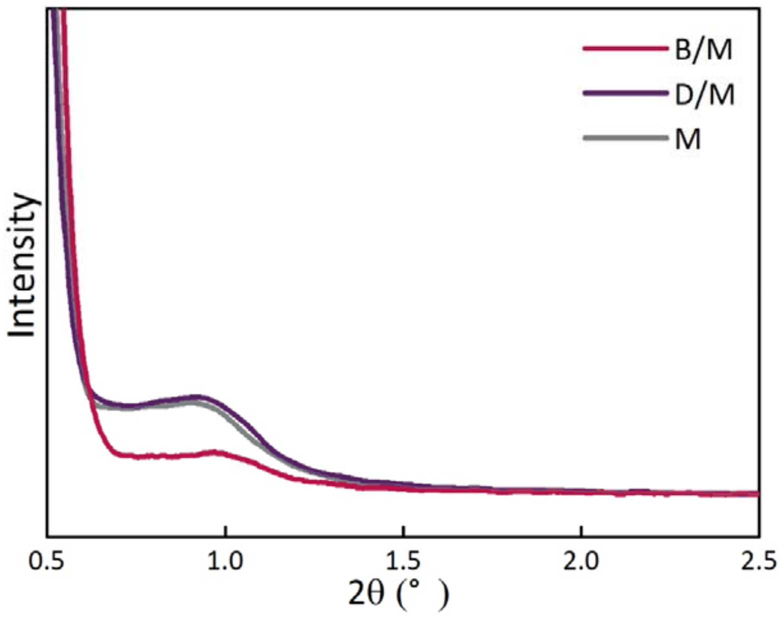

Figure S1. SAXRD patterns of B/M, D/M and M.

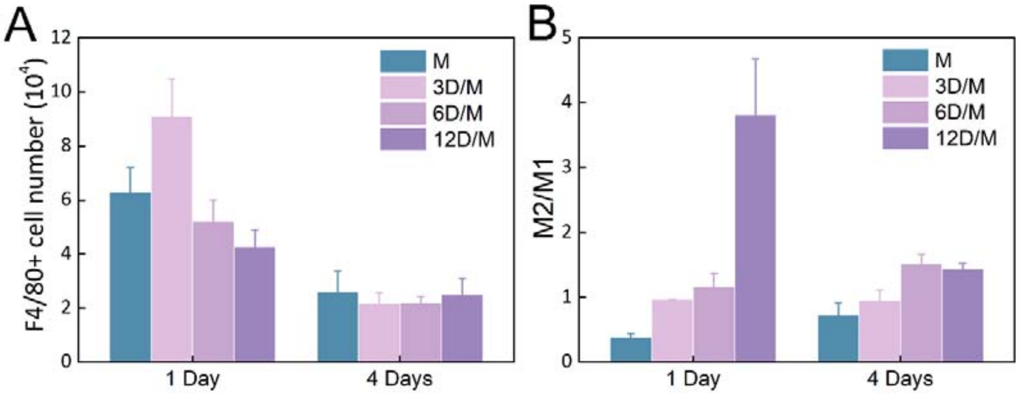

Figure S2. Penetrating F4/80+ monocytes/macrophages to implanted scaffolds with different dose of Dex at 1 and 4 days. (A) Cell number of F4/80+ monocytes/macrophages. (B) The ratio of M2/M1.

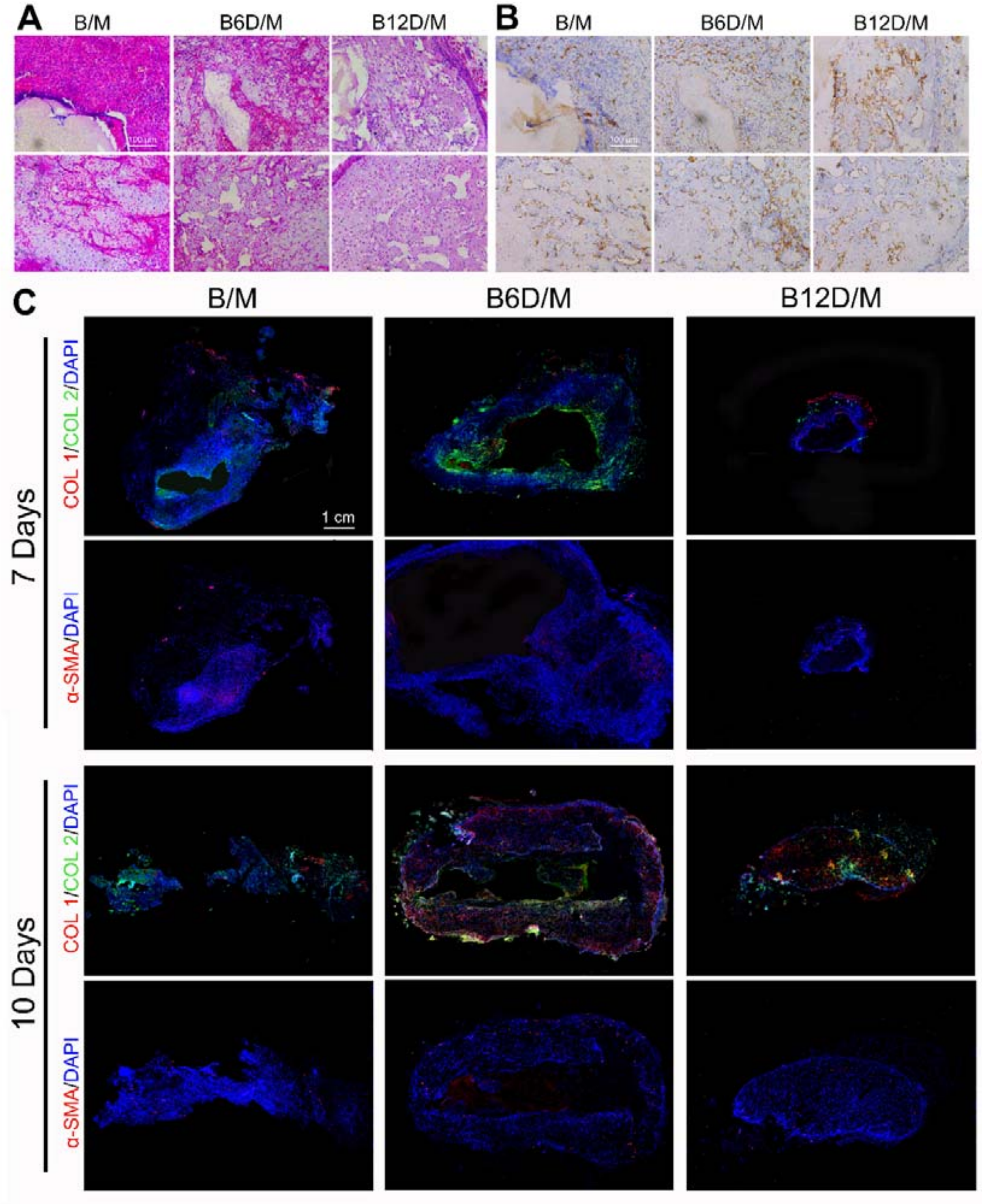

Figure S3. (A) HE staining at 10 days. The upper row is the section close to implanted scaffolds, and the under row is the distal section. (B) Immunohistochemistry analyses of CD31 expression at 10 days. The upper row is the section close to implanted scaffolds, and the under row is the distal section. (C) Complete pictures of immunofluorescent images.

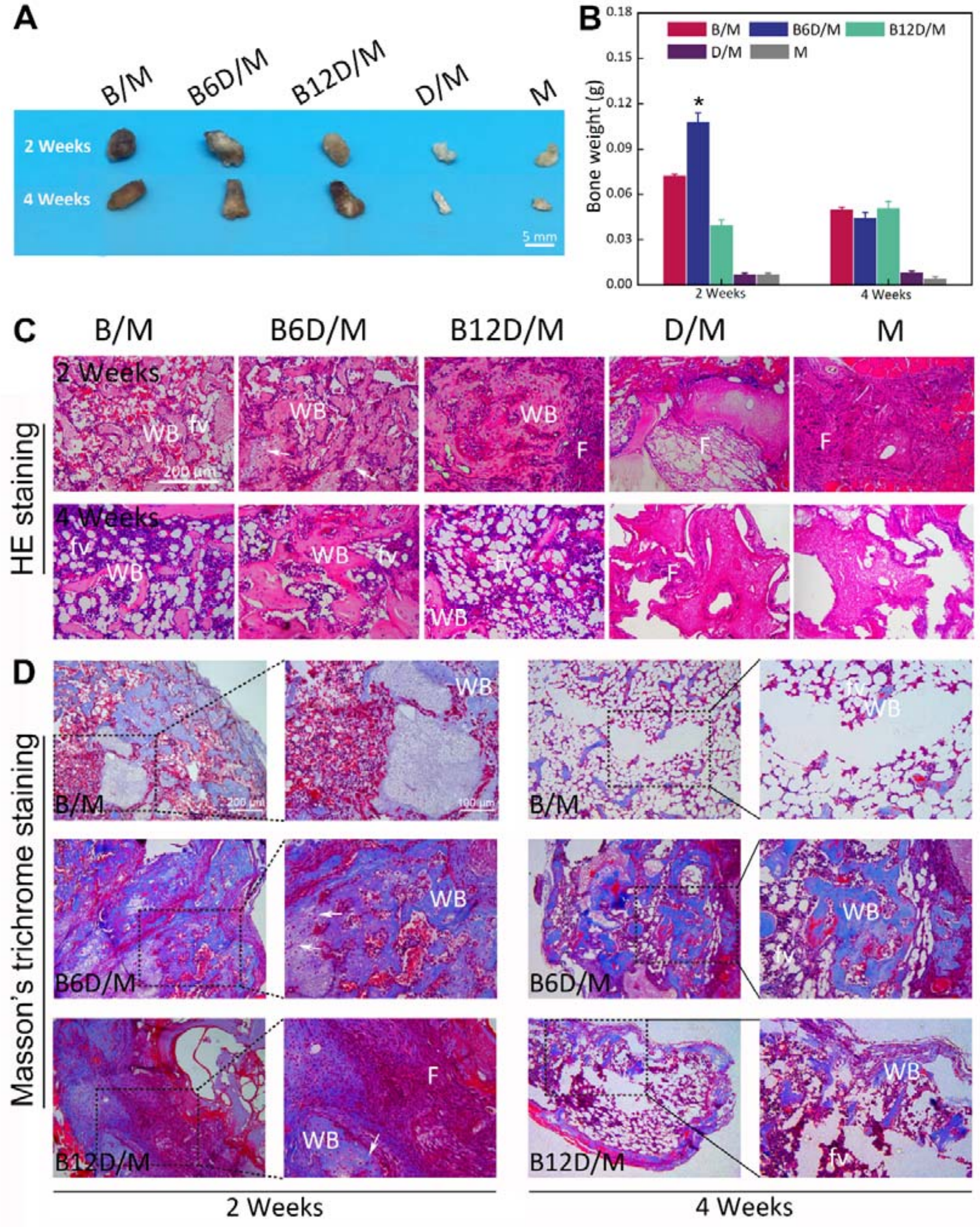

Figure S4. (A) Digital images of ectopic bones at 2 and 4 weeks. (B) Quantification of bone weight. (C) HE staining. (D) Masson's trichrome staining. (Woven bone: WB; fat vacuoles: fv; fibrous: F; hypertrophic chondrocytes: white arrow.). (*$p < 0.05$, N = 3.)

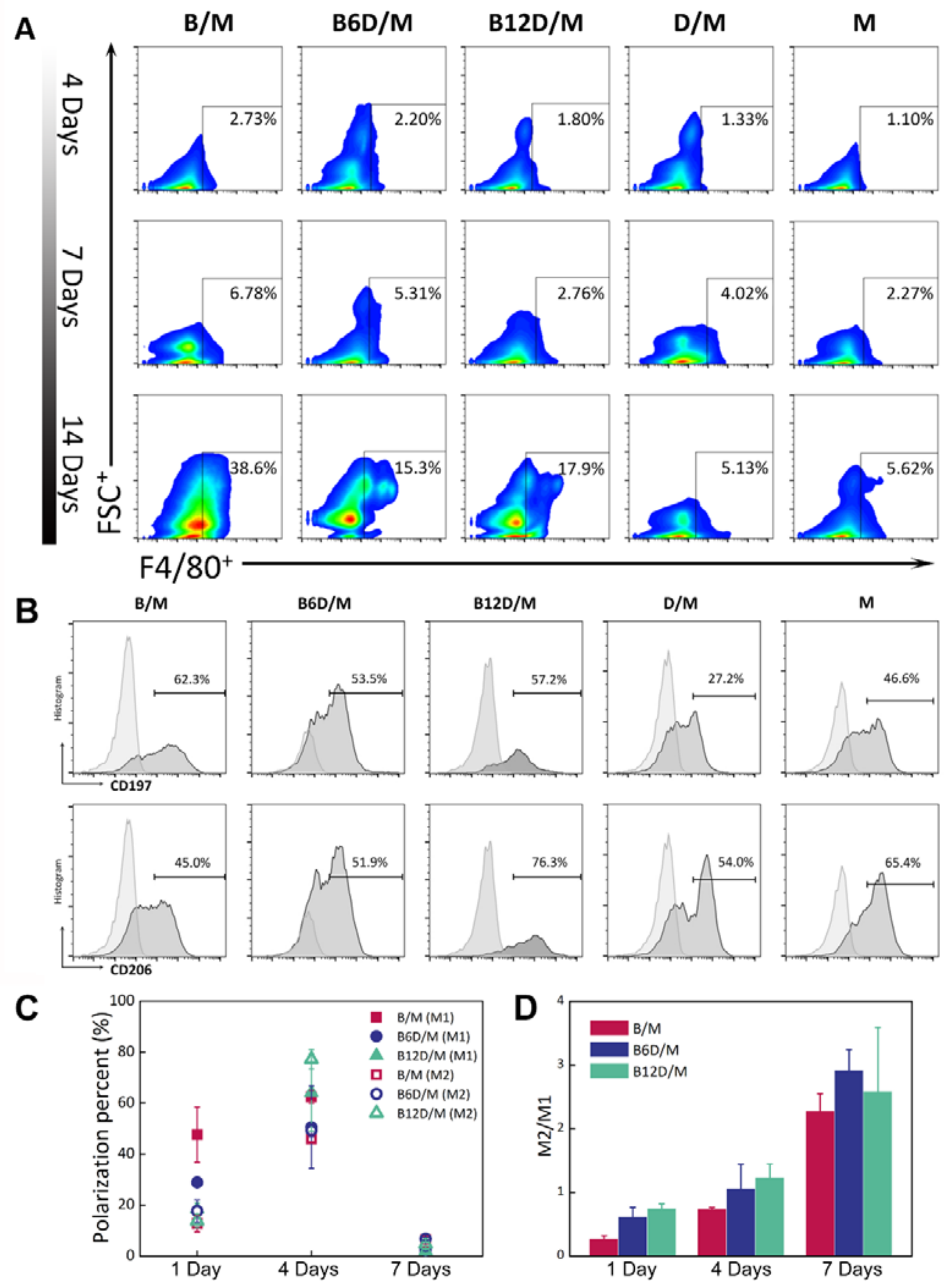

Figure S5. Analysis of F4/80+ macrophages/monocytes recruitment at days 4, 7 and 14 (A), and M1 phenotype and M2 phenotype at day 4 by flow cytometry (B). (C) Percentages of M1 phenotype and M2 phenotype at 1, 4, 7 days. (D) Ratio of M2/M1 at 1, 4, 7 days.

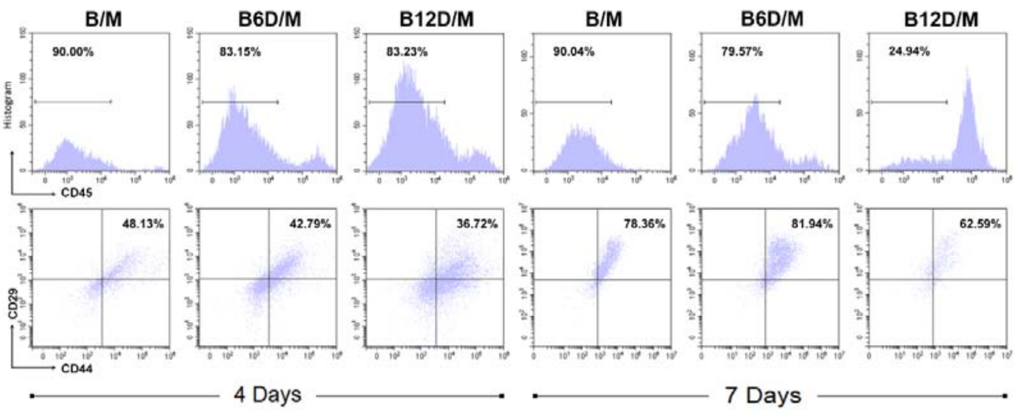

Figure S6. Analysis of MSCs recruitment of B/M, B6D/M and B12D/M at 4 days and 7 days by flow cytometry.

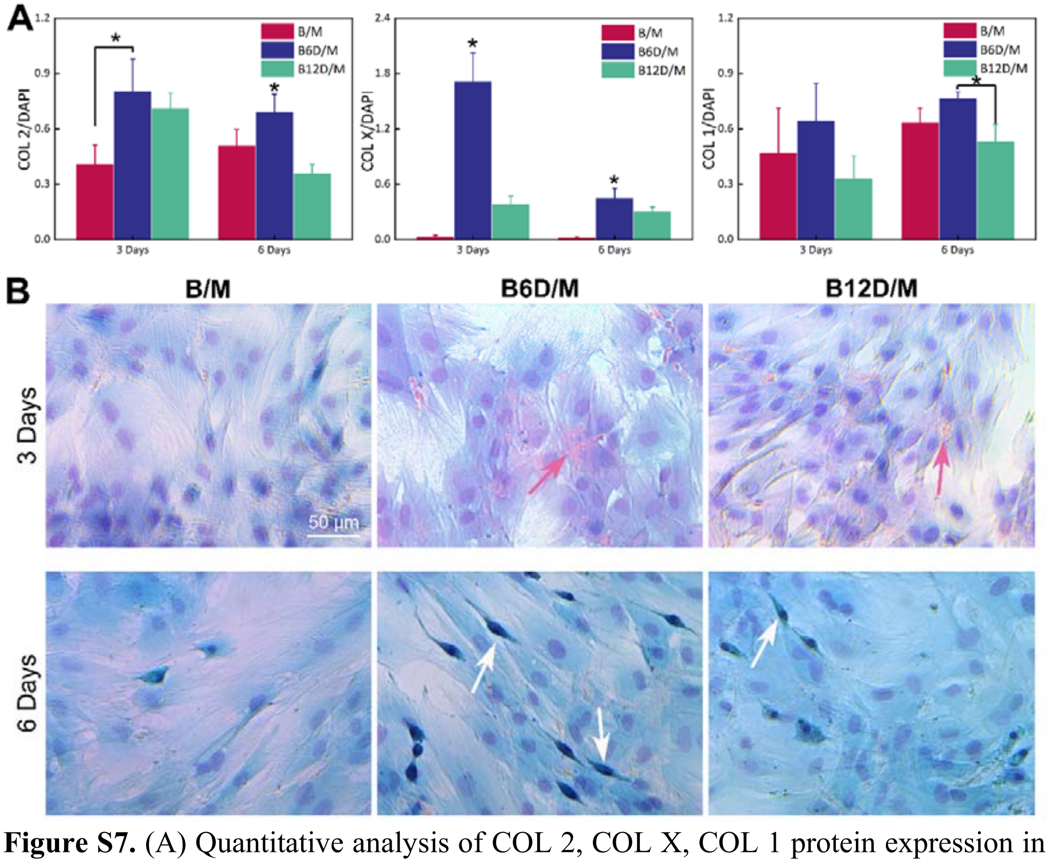

Figure S7. (A) Quantitative analysis of COL 2, COL X, COL 1 protein expression in Figure 4E by Image J. (B) Safranin-O-fast green staining of MSCs at 3 days and 6 days after cultured with retrieved samples. (Aggrecan: pink arrow; osteoblasts: white arrow.) (*$p < 0.05$, N = 3.)

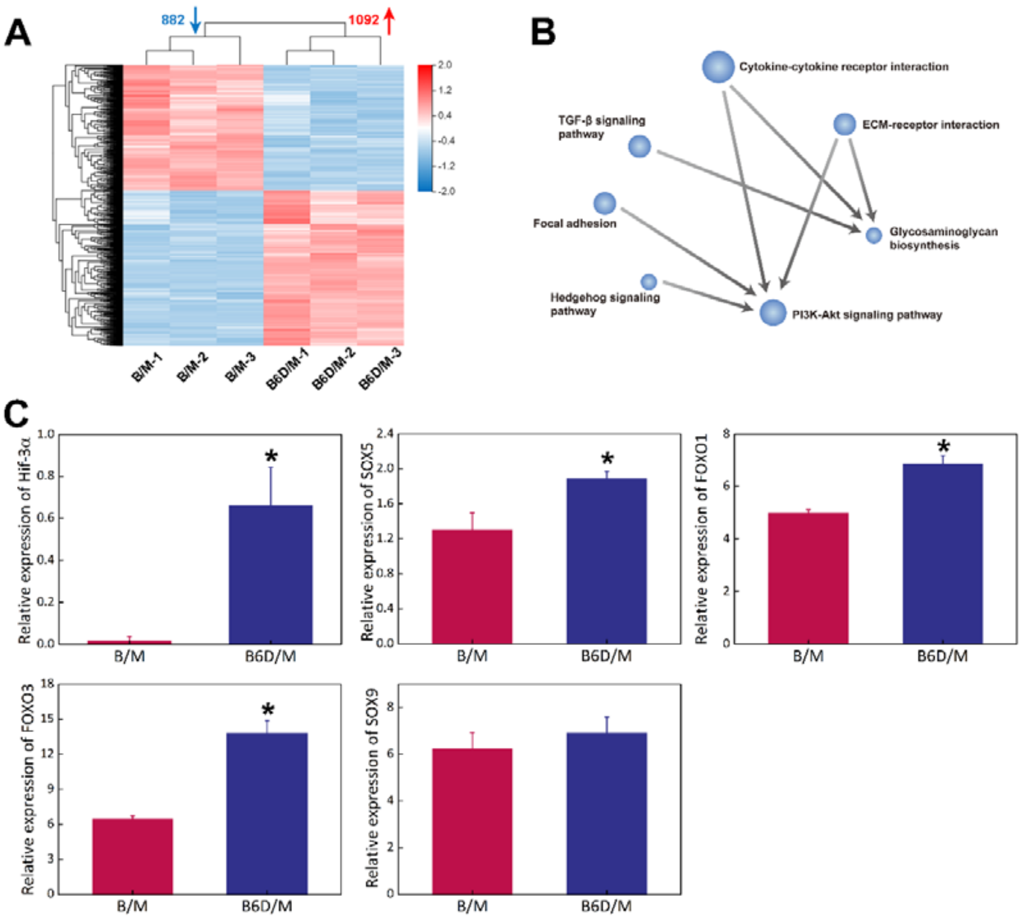

Figure S8. (A) A heatmap of differentially expressed transcripts. (B) Interactive network of chondrogenic signaling pathways. (C) Relative gene expression of Hif-3α, SOX5, FOXO1, FOXO3 and SOX9. (*$p < 0.05$, N = 3.)

Table S1. Primer sequences used in RT-qPCR.

Gene	Direction	Sequence(5'-3')
COMP	Forward	TGACTTCGATGCTGACAAGG
	Reverse	GAACGATCTCCATTCCCTGA
COL 2	Forward	CCCCTGCAGTACATGCGG
	Reverse	CTCGACGTCATGCTGTCTCAAG
ALP	Forward	CTCCGGATCCTGACAAAGAA
	Reverse	ACGTGGGGGATGTAGTTCTG

Runx2	Forward	CGGCCCTCCCTGAACTCT
	Reverse	TGCCTGCCTGGGATCTGT
COL 1	Forward	GGTATGCTTGATCTGTATCTGC
	Reverse	AGTCCAGTTCTTCATTGCATT
GAPDH	Forward	CCCCCAATGTATCCGTTGTG
	Reverse	TAGCCCAGGATGCCCTTTAGT
